# 5-[(1-Benzyl-1*H*-1,2,3-triazol-4-yl)meth­yl]-5*H*-dibenzo[*b,f*]azepine

**DOI:** 10.1107/S1600536813030547

**Published:** 2013-11-13

**Authors:** B. C. Manjunath, K. S. Vinay Kumar, S. Madan Kumar, M. P. Sadashiva, N. K. Lokanath

**Affiliations:** a Department of Studies in Physics, Manasagangotri, University of Mysore, Mysore 570 006, India; b Department of Studies in Chemistry, Manasagangotri, University of Mysore, Mysore 570 006, India

## Abstract

In the title compound, C_24_H_20_N_4_, the azepine ring adopts a boat conformation. The dihedral angle between the benzene rings fused to the azepine ring is 49.40 (9)°. The triazole ring makes a dihedral angle of 77.88 (9)° with the terminal phenyl ring. In the crystal, mol­ecules are linked *via* C—H⋯π inter­actions and a parallel slipped π–π inter­action [centroid–centroid distance = 3.7324 (9), normal distance = 3.4060 (6) and slippage = 1.526 Å], forming a three-dimensional network.

## Related literature

For the use of dibenzo azepine derivatives in the preparation of carbamazepine, see: Rockliff & Davis (1966 ▶). For their anti­tumor properties, see: Al-Qawasmeh *et al.* (2009 ▶). For related structures, see: Abdoh *et al.* (2013 ▶); Manjunath *et al.* (2013 ▶). For ring-puckering analysis, see: Cremer & Pople (1975 ▶).
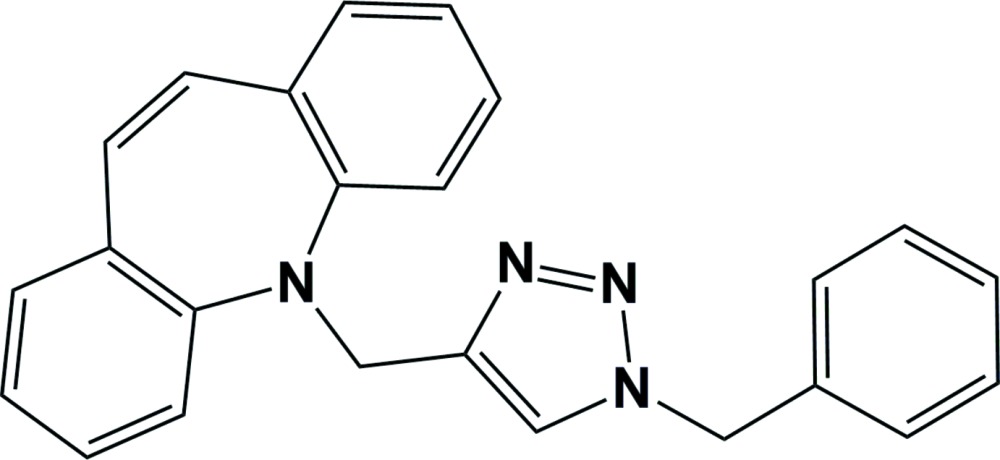


## Experimental

### 

#### Crystal data

C_24_H_20_N_4_
*M* *_r_* = 364.44
Monoclinic, 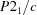

*a* = 11.4339 (10) Å
*b* = 11.7140 (12) Å
*c* = 14.4527 (13) Å
β = 98.610 (4)°
*V* = 1913.9 (3) Å^3^
*Z* = 4
Cu *K*α radiation
μ = 0.60 mm^−1^
*T* = 296 K
0.23 × 0.22 × 0.21 mm

#### Data collection

Bruker X8 Proteum diffractometer
Absorption correction: multi-scan (*SADABS*; Bruker, 2013 ▶) *T*_min_ = 0.871, *T*_max_ = 0.882
11434 measured reflections
3160 independent reflections
2742 reflections with *I* > 2σ(*I*)
*R*_int_ = 0.029

#### Refinement

*R*[*F* ^2^ > 2σ(*F* ^2^)] = 0.047
*wR*(*F* ^2^) = 0.135
*S* = 1.06
3160 reflections
242 parameters
H-atom parameters constrained
Δρ_max_ = 0.37 e Å^−3^
Δρ_min_ = −0.35 e Å^−3^

### 

Data collection: *APEX2* (Bruker, 2013 ▶); cell refinement: *SAINT* (Bruker, 2013 ▶); data reduction: *SAINT*; program(s) used to solve structure: *SHELXS97* (Sheldrick, 2008 ▶); program(s) used to refine structure: *SHELXL97* (Sheldrick, 2008 ▶); molecular graphics: *Mercury* (Macrae *et al.*, 2008 ▶); software used to prepare material for publication: *Mercury* (Macrae *et al.*, 2008 ▶).

## Supplementary Material

Crystal structure: contains datablock(s) global, I. DOI: 10.1107/S1600536813030547/su2664sup1.cif

Structure factors: contains datablock(s) I. DOI: 10.1107/S1600536813030547/su2664Isup2.hkl

Supplementary material file. DOI: 10.1107/S1600536813030547/su2664Isup3.cml

Additional supplementary materials:  crystallographic information; 3D view; checkCIF report

## Figures and Tables

**Table 1:** Hydrogen-bond geometry (Å, °) *Cg*1 and *Cg*2 are the centroids of the C1–C6 and C9–C14 rings, respectively

*D*—H⋯*A*	*D*—H	H⋯*A*	*D*⋯*A*	*D*—H⋯*A*
C18—H18*A*⋯*Cg*2^i^	0.97	2.83	3.600 (2)	137
C20—H20⋯*Cg*1^i^	0.93	2.79	3.642 (2)	153

Symmetry code: (i) 
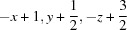
.

**Fig. 1.**
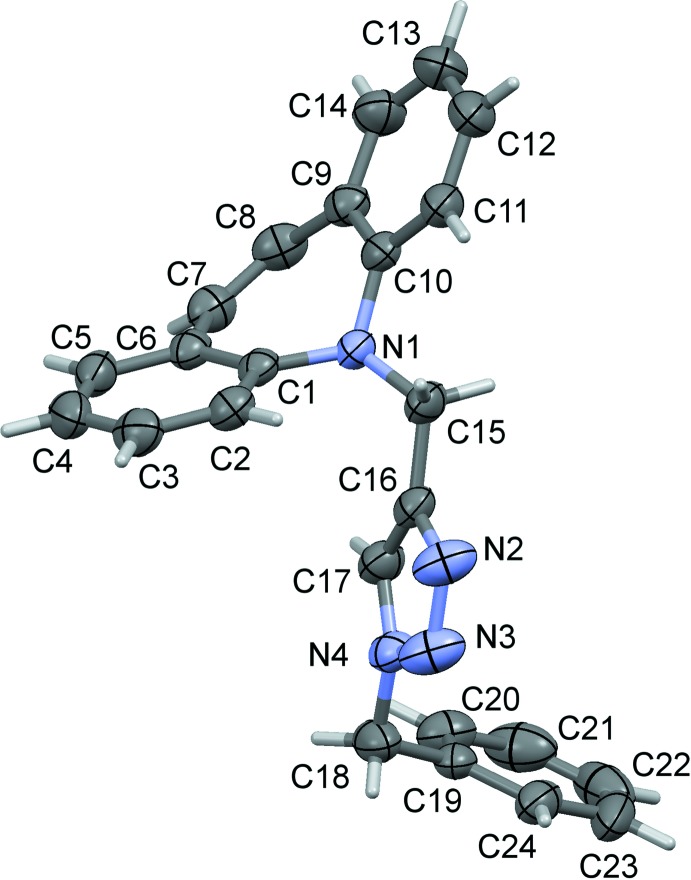
A view of the molecular structure of the title molecule, with atom labelling. Displacement ellipsoids are drawn at the 50% probability level.

**Fig. 2.**
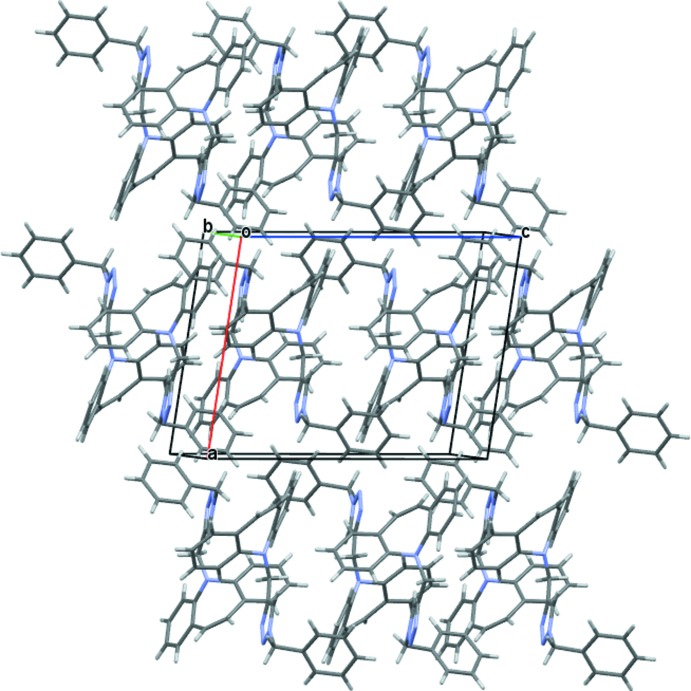
A viewed along the b axis of the crystal packing of the title compound.

## supplementary crystallographic information

### 1. Comment

Dibenzo azepine derivatives have been shown to act as antitumor drugs (Al-Qawasmeh *et al.*, 2009). They are used in the prepration of carbamazepine, an anticonvulsant (Rockliff & Davis, 1966). As a part of our on-going research on the synthesis and crystal structural studies of dibenzo azepine derivatives (Abdoh *et al.*, 2013; Manjunath *et al.*, 2013), we present herein the crystal structure of the title compound.

The molecular structure of the title molecule is shown in Fig. 1. The seven-membered azepine ring adopts a boat conformation with puckering parameters (Cremer & Pople, 1975): Q_2_ = 0.6845 (18) Å, Q3 = 0.2069 (17) Å, φ_2_ = 178.28 (16)°, φ_3_ = 178.9 (5)°, and a total puckering amplitude Q_T_ = 0.7149 (17) Å .

The dihedral angle between the two benzene rings, (C1—C6) and (C9—C14), fused to the azepine ring is 49.40 (9)°. The triazole ring (C16/N2—N4/C17) makes a dihedral angle of 77.88 (9)° with the terminal phenyl ring (C19—C24). The overall geometry of the title molecule is similar that of earlier reported structures (Abdoh *et al.*, 2013; Manjunath *et al.*, 2013).

In the crystal, molecules are connected by C—H···π interactions (Table 1), and a slipped parallel π-π interaction involving inversion related terminal phenyl rings [*Cg3*···*Cg3*^i^ = 3.7324 (9) Å; normal distance = 3.4060 (6) Å; slippage = 1.526 Å; *Cg3* is the centroid of ring (C20—C24); symmetry code: (i) = -*x* + 2, -*y* + 1, -*z* + 2]. These interactions result in the formation of a three-dimensional structure (Fig. 2).

### 2. Experimental

5-(prop-2-yn-1-yl)-5*H*-dibenzo[*b,f*]azepine (2.1 mmol) was taken in a mixture of dichloromethane and water in the ratio 1:1, Cuprous iodide (0.21 mmol) was added followed by Sodium ascorbate (0.21 mmol) at room temperature. After 10 minutes, benzyl azide was added (2.3 mmol) at room temperature. The resulting reaction mixture was stirred for 6 h. After completion of the reaction (monitored by TLC), the reaction mixture was diluted with water (50 ml). The aqueous layer was extracted with ethyl acetate (3 × 20 ml), the combined ethyl acetate layer was washed with brine solution (2 × 25 ml). The organic layer was then dried over anhydrous sodium sulfate, filtered and concentrated under reduced pressure. The crude product obtained was purified by column chromatography over silica gel (60–120 mesh) using hexane:ethyl acetate (8:2) as eluent. The pure compound was recrystallized in ethyl acetate/hexane (1:1) to obtain light-yellow block-like crystals.

### 3. Refinement

All the H atoms were fixed geometrically and allowed to ride on their parent atoms: C—H= 0.93–0.97 Å with *U*_iso_(H) = 1.2*U*_eq_(C). The benzene ring (C19-C24) was refined as a regular hexagon.

### Crystal data

**Table:**

C_24_H_20_N_4_	*F*(000) = 768
*M**_r_* = 364.44	*D*_x_ = 1.265 Mg m^−^^3^
Monoclinic, *P*2_1_/*c*	Cu *K*α radiation, λ = 1.54178 Å
Hall symbol: -P 2ybc	Cell parameters from 3160 reflections
*a* = 11.4339 (10) Å	θ = 3.9–64.7°
*b* = 11.7140 (12) Å	µ = 0.60 mm^−^^1^
*c* = 14.4527 (13) Å	*T* = 296 K
β = 98.610 (4)°	Block, yellow
*V* = 1913.9 (3) Å^3^	0.23 × 0.22 × 0.21 mm
*Z* = 4	

### Data collection

**Table:**

Bruker X8 Proteum diffractometer	3160 independent reflections
Radiation source: Bruker MicroStar microfocus rotating anode	2742 reflections with *I* > 2σ(*I*)
Helios multilayer optics monochromator	*R*_int_ = 0.029
Detector resolution: 10.7 pixels mm^-1^	θ_max_ = 64.7°, θ_min_ = 3.9°
φ and ω scans	*h* = −13→13
Absorption correction: multi-scan (*SADABS*; Bruker, 2013)	*k* = −6→13
*T*_min_ = 0.871, *T*_max_ = 0.882	*l* = −16→16
11434 measured reflections	

### Refinement

**Table:**

Refinement on *F*^2^	Secondary atom site location: difference Fourier map
Least-squares matrix: full	Hydrogen site location: inferred from neighbouring sites
*R*[*F*^2^ > 2σ(*F*^2^)] = 0.047	H-atom parameters constrained
*wR*(*F*^2^) = 0.135	*w* = 1/[σ^2^(*F*_o_^2^) + (0.0672*P*)^2^ + 0.668*P*] where *P* = (*F*_o_^2^ + 2*F*_c_^2^)/3
*S* = 1.06	(Δ/σ)_max_ < 0.001
3160 reflections	Δρ_max_ = 0.37 e Å^−^^3^
242 parameters	Δρ_min_ = −0.35 e Å^−^^3^
0 restraints	Extinction correction: *SHELXL97* (Sheldrick, 2008), Fc^*^=kFc[1+0.001xFc^2^λ^3^/sin(2θ)]^-1/4^
Primary atom site location: structure-invariant direct methods	Extinction coefficient: 0.0022 (4)

### Special details

**Table:**

Geometry. Bond distances, angles etc. have been calculated using the rounded fractional coordinates. All su's are estimated from the variances of the (full) variance-covariance matrix. The cell esds are taken into account in the estimation of distances, angles and torsion angles
Refinement. Refinement of F^2^ against ALL reflections. The weighted R-factor wR and goodness of fit S are based on F^2^, conventional R-factors R are based on F, with F set to zero for negative F^2^. The threshold expression of F^2^ > 2sigma(F^2^) is used only for calculating R-factors(gt) etc. and is not relevant to the choice of reflections for refinement. R-factors based on F^2^ are statistically about twice as large as those based on F, and R- factors based on ALL data will be even larger.

### Fractional atomic coordinates and isotropic or equivalent isotropic displacement parameters (Å^2^)

**Table:**

	*x*	*y*	*z*	*U*_iso_*/*U*_eq_	
N1	0.43569 (11)	0.09444 (13)	0.81026 (9)	0.0380 (4)	
N2	0.75967 (14)	0.07129 (16)	0.84538 (13)	0.0587 (6)	
N3	0.83816 (14)	0.15341 (17)	0.84788 (13)	0.0615 (6)	
N4	0.78072 (13)	0.25199 (14)	0.84990 (10)	0.0466 (5)	
C1	0.42570 (14)	0.13226 (14)	0.71500 (11)	0.0362 (5)	
C2	0.50035 (15)	0.09093 (17)	0.65474 (12)	0.0444 (6)	
C3	0.49640 (18)	0.13523 (19)	0.56554 (13)	0.0529 (7)	
C4	0.41693 (18)	0.22002 (19)	0.53381 (13)	0.0552 (7)	
C5	0.34204 (17)	0.26084 (17)	0.59263 (13)	0.0493 (6)	
C6	0.34446 (15)	0.21923 (15)	0.68381 (12)	0.0397 (5)	
C7	0.26469 (16)	0.26982 (16)	0.74219 (13)	0.0472 (6)	
C8	0.21604 (16)	0.21843 (17)	0.80959 (14)	0.0502 (6)	
C9	0.22998 (15)	0.10011 (17)	0.83926 (12)	0.0434 (6)	
C10	0.33367 (14)	0.03702 (16)	0.83579 (11)	0.0390 (5)	
C11	0.33787 (16)	−0.07699 (17)	0.86185 (13)	0.0486 (6)	
C12	0.24221 (19)	−0.1295 (2)	0.89303 (15)	0.0606 (8)	
C13	0.14115 (19)	−0.0676 (2)	0.89868 (16)	0.0654 (8)	
C14	0.13561 (18)	0.0451 (2)	0.87236 (14)	0.0577 (7)	
C15	0.54828 (15)	0.04203 (17)	0.85030 (13)	0.0450 (6)	
C16	0.65211 (14)	0.11824 (16)	0.84764 (11)	0.0404 (6)	
C17	0.66473 (15)	0.23304 (17)	0.85105 (13)	0.0473 (6)	
C18	0.84716 (19)	0.3590 (2)	0.86075 (13)	0.0559 (7)	
C19	0.86839 (10)	0.39520 (10)	0.96072 (6)	0.0391 (5)	
C20	0.78941 (10)	0.47031 (11)	0.99293 (9)	0.0577 (7)	
C21	0.80418 (14)	0.50082 (13)	1.08690 (11)	0.0767 (10)	
C22	0.89793 (16)	0.45624 (15)	1.14866 (7)	0.0805 (10)	
C23	0.97691 (13)	0.38113 (14)	1.11645 (8)	0.0791 (9)	
C24	0.96214 (10)	0.35062 (11)	1.02248 (9)	0.0527 (7)	
H2	0.55350	0.03280	0.67480	0.0530*	
H3	0.54780	0.10750	0.52670	0.0630*	
H4	0.41380	0.24930	0.47370	0.0660*	
H5	0.28820	0.31780	0.57110	0.0590*	
H7	0.24560	0.34630	0.73130	0.0570*	
H8	0.16780	0.26350	0.84110	0.0600*	
H11	0.40610	−0.11910	0.85840	0.0580*	
H12	0.24630	−0.20610	0.91000	0.0730*	
H13	0.07700	−0.10200	0.92020	0.0790*	
H14	0.06700	0.08620	0.87660	0.0690*	
H15A	0.55930	−0.02740	0.81610	0.0540*	
H15B	0.54470	0.02110	0.91480	0.0540*	
H17	0.60590	0.28730	0.85360	0.0570*	
H18A	0.80330	0.41800	0.82330	0.0670*	
H18B	0.92240	0.34900	0.83840	0.0670*	
H20	0.72670	0.50010	0.95160	0.0690*	
H21	0.75130	0.55110	1.10850	0.0920*	
H22	0.90780	0.47670	1.21150	0.0970*	
H23	1.03960	0.35130	1.15780	0.0950*	
H24	1.01500	0.30040	1.00090	0.0630*	

### Atomic displacement parameters (Å^2^)

**Table:**

	*U*^11^	*U*^22^	*U*^33^	*U*^12^	*U*^13^	*U*^23^
N1	0.0325 (7)	0.0412 (8)	0.0377 (7)	−0.0023 (6)	−0.0028 (5)	0.0018 (6)
N2	0.0383 (8)	0.0599 (11)	0.0768 (12)	−0.0014 (8)	0.0054 (8)	−0.0219 (9)
N3	0.0411 (9)	0.0662 (12)	0.0775 (12)	−0.0055 (8)	0.0101 (8)	−0.0265 (9)
N4	0.0411 (8)	0.0578 (10)	0.0396 (8)	−0.0123 (7)	0.0016 (6)	−0.0084 (7)
C1	0.0342 (8)	0.0356 (9)	0.0361 (8)	−0.0059 (7)	−0.0032 (6)	−0.0037 (7)
C2	0.0399 (9)	0.0466 (11)	0.0447 (10)	−0.0010 (8)	−0.0001 (7)	−0.0063 (8)
C3	0.0518 (11)	0.0648 (14)	0.0422 (10)	−0.0080 (10)	0.0078 (8)	−0.0096 (9)
C4	0.0608 (12)	0.0639 (14)	0.0385 (10)	−0.0142 (10)	−0.0008 (9)	0.0034 (9)
C5	0.0500 (10)	0.0450 (11)	0.0478 (10)	−0.0040 (8)	−0.0091 (8)	0.0054 (8)
C6	0.0386 (9)	0.0344 (9)	0.0431 (9)	−0.0048 (7)	−0.0035 (7)	−0.0015 (7)
C7	0.0455 (10)	0.0356 (10)	0.0584 (11)	0.0037 (8)	0.0011 (8)	−0.0026 (8)
C8	0.0419 (10)	0.0499 (12)	0.0589 (11)	0.0036 (8)	0.0079 (8)	−0.0119 (9)
C9	0.0395 (9)	0.0497 (11)	0.0399 (9)	−0.0034 (8)	0.0028 (7)	−0.0061 (8)
C10	0.0374 (9)	0.0445 (11)	0.0329 (8)	−0.0062 (7)	−0.0018 (6)	−0.0020 (7)
C11	0.0439 (10)	0.0497 (12)	0.0490 (10)	−0.0051 (8)	−0.0038 (8)	0.0070 (8)
C12	0.0601 (13)	0.0582 (14)	0.0602 (12)	−0.0146 (10)	−0.0019 (10)	0.0169 (10)
C13	0.0521 (12)	0.0800 (17)	0.0652 (13)	−0.0177 (11)	0.0120 (10)	0.0137 (12)
C14	0.0428 (11)	0.0732 (15)	0.0582 (12)	−0.0036 (10)	0.0108 (9)	0.0013 (10)
C15	0.0368 (9)	0.0465 (11)	0.0478 (10)	−0.0007 (8)	−0.0066 (7)	0.0048 (8)
C16	0.0347 (9)	0.0487 (11)	0.0351 (9)	−0.0011 (7)	−0.0040 (7)	−0.0034 (7)
C17	0.0356 (9)	0.0521 (12)	0.0521 (10)	0.0000 (8)	−0.0006 (8)	0.0013 (9)
C18	0.0566 (12)	0.0647 (14)	0.0451 (10)	−0.0223 (10)	0.0032 (9)	0.0003 (9)
C19	0.0352 (8)	0.0382 (10)	0.0422 (9)	−0.0097 (7)	0.0005 (7)	0.0027 (7)
C20	0.0457 (11)	0.0518 (13)	0.0753 (14)	−0.0038 (9)	0.0080 (9)	−0.0076 (10)
C21	0.0734 (16)	0.0795 (18)	0.0832 (17)	−0.0254 (14)	0.0317 (14)	−0.0304 (14)
C22	0.107 (2)	0.088 (2)	0.0506 (13)	−0.0487 (17)	0.0250 (13)	−0.0149 (13)
C23	0.0800 (16)	0.0812 (18)	0.0630 (14)	−0.0326 (14)	−0.0319 (12)	0.0246 (13)
C24	0.0437 (10)	0.0481 (12)	0.0618 (12)	−0.0041 (8)	−0.0064 (9)	0.0076 (9)

### Geometric parameters (Å, º)

**Table:**

N1—C1	1.435 (2)	C19—C20	1.3900 (17)
N1—C10	1.442 (2)	C19—C24	1.3900 (16)
N1—C15	1.465 (2)	C20—C21	1.390 (2)
N2—N3	1.312 (3)	C21—C22	1.390 (2)
N2—C16	1.352 (2)	C22—C23	1.390 (2)
N3—N4	1.331 (2)	C23—C24	1.3900 (18)
N4—C17	1.347 (2)	C2—H2	0.9300
N4—C18	1.462 (3)	C3—H3	0.9300
C1—C2	1.394 (2)	C4—H4	0.9300
C1—C6	1.407 (2)	C5—H5	0.9300
C2—C3	1.384 (3)	C7—H7	0.9300
C3—C4	1.378 (3)	C8—H8	0.9300
C4—C5	1.379 (3)	C11—H11	0.9300
C5—C6	1.401 (3)	C12—H12	0.9300
C6—C7	1.458 (3)	C13—H13	0.9300
C7—C8	1.335 (3)	C14—H14	0.9300
C8—C9	1.453 (3)	C15—H15A	0.9700
C9—C10	1.404 (2)	C15—H15B	0.9700
C9—C14	1.401 (3)	C17—H17	0.9300
C10—C11	1.387 (3)	C18—H18A	0.9700
C11—C12	1.388 (3)	C18—H18B	0.9700
C12—C13	1.377 (3)	C20—H20	0.9300
C13—C14	1.373 (3)	C21—H21	0.9300
C15—C16	1.491 (3)	C22—H22	0.9300
C16—C17	1.353 (3)	C23—H23	0.9300
C18—C19	1.490 (2)	C24—H24	0.9300
			
C1—N1—C10	116.01 (13)	C19—C24—C23	120.00 (12)
C1—N1—C15	116.59 (13)	C1—C2—H2	119.00
C10—N1—C15	113.59 (14)	C3—C2—H2	120.00
N3—N2—C16	108.78 (17)	C2—C3—H3	120.00
N2—N3—N4	107.39 (15)	C4—C3—H3	120.00
N3—N4—C17	110.32 (16)	C3—C4—H4	121.00
N3—N4—C18	119.74 (16)	C5—C4—H4	120.00
C17—N4—C18	129.54 (17)	C4—C5—H5	119.00
N1—C1—C2	121.60 (15)	C6—C5—H5	119.00
N1—C1—C6	119.02 (14)	C6—C7—H7	116.00
C2—C1—C6	119.21 (15)	C8—C7—H7	116.00
C1—C2—C3	120.93 (17)	C7—C8—H8	116.00
C2—C3—C4	120.55 (18)	C9—C8—H8	116.00
C3—C4—C5	118.96 (18)	C10—C11—H11	119.00
C4—C5—C6	122.16 (18)	C12—C11—H11	119.00
C1—C6—C5	118.19 (16)	C11—C12—H12	120.00
C1—C6—C7	123.32 (16)	C13—C12—H12	120.00
C5—C6—C7	118.48 (16)	C12—C13—H13	120.00
C6—C7—C8	127.16 (18)	C14—C13—H13	120.00
C7—C8—C9	127.32 (18)	C9—C14—H14	119.00
C8—C9—C10	123.20 (16)	C13—C14—H14	119.00
C8—C9—C14	118.92 (17)	N1—C15—H15A	109.00
C10—C9—C14	117.88 (18)	N1—C15—H15B	109.00
N1—C10—C9	118.91 (16)	C16—C15—H15A	109.00
N1—C10—C11	121.50 (15)	C16—C15—H15B	109.00
C9—C10—C11	119.49 (16)	H15A—C15—H15B	108.00
C10—C11—C12	121.22 (18)	N4—C17—H17	127.00
C11—C12—C13	119.7 (2)	C16—C17—H17	127.00
C12—C13—C14	119.6 (2)	N4—C18—H18A	110.00
C9—C14—C13	122.1 (2)	N4—C18—H18B	109.00
N1—C15—C16	113.27 (16)	C19—C18—H18A	109.00
N2—C16—C15	119.20 (17)	C19—C18—H18B	109.00
N2—C16—C17	108.23 (16)	H18A—C18—H18B	108.00
C15—C16—C17	132.50 (16)	C19—C20—H20	120.00
N4—C17—C16	105.26 (16)	C21—C20—H20	120.00
N4—C18—C19	110.77 (15)	C20—C21—H21	120.00
C18—C19—C20	119.02 (12)	C22—C21—H21	120.00
C18—C19—C24	120.91 (13)	C21—C22—H22	120.00
C20—C19—C24	120.00 (10)	C23—C22—H22	120.00
C19—C20—C21	120.00 (12)	C22—C23—H23	120.00
C20—C21—C22	120.00 (14)	C24—C23—H23	120.00
C21—C22—C23	120.00 (11)	C19—C24—H24	120.00
C22—C23—C24	120.00 (12)	C23—C24—H24	120.00
			
C10—N1—C1—C2	−118.27 (18)	C1—C6—C7—C8	−32.0 (3)
C10—N1—C1—C6	66.5 (2)	C5—C6—C7—C8	149.4 (2)
C15—N1—C1—C2	19.7 (2)	C6—C7—C8—C9	−1.0 (3)
C15—N1—C1—C6	−155.60 (16)	C7—C8—C9—C10	31.6 (3)
C1—N1—C10—C9	−68.6 (2)	C7—C8—C9—C14	−147.9 (2)
C1—N1—C10—C11	114.98 (18)	C8—C9—C10—N1	6.2 (3)
C15—N1—C10—C9	152.20 (15)	C8—C9—C10—C11	−177.34 (17)
C15—N1—C10—C11	−24.2 (2)	C14—C9—C10—N1	−174.34 (16)
C1—N1—C15—C16	57.8 (2)	C14—C9—C10—C11	2.1 (3)
C10—N1—C15—C16	−163.24 (14)	C8—C9—C14—C13	177.84 (19)
C16—N2—N3—N4	−1.2 (2)	C10—C9—C14—C13	−1.7 (3)
N3—N2—C16—C15	−176.98 (16)	N1—C10—C11—C12	175.13 (17)
N3—N2—C16—C17	0.4 (2)	C9—C10—C11—C12	−1.2 (3)
N2—N3—N4—C17	1.5 (2)	C10—C11—C12—C13	−0.2 (3)
N2—N3—N4—C18	174.94 (16)	C11—C12—C13—C14	0.8 (3)
N3—N4—C17—C16	−1.3 (2)	C12—C13—C14—C9	0.2 (3)
C18—N4—C17—C16	−173.84 (16)	N1—C15—C16—N2	−153.38 (16)
N3—N4—C18—C19	−95.86 (19)	N1—C15—C16—C17	30.0 (3)
C17—N4—C18—C19	76.1 (2)	N2—C16—C17—N4	0.5 (2)
N1—C1—C2—C3	−174.45 (17)	C15—C16—C17—N4	177.41 (17)
C6—C1—C2—C3	0.8 (3)	N4—C18—C19—C20	−93.75 (18)
N1—C1—C6—C5	175.42 (16)	N4—C18—C19—C24	83.06 (19)
N1—C1—C6—C7	−3.2 (3)	C18—C19—C20—C21	176.83 (15)
C2—C1—C6—C5	0.1 (2)	C24—C19—C20—C21	−0.02 (19)
C2—C1—C6—C7	−178.55 (17)	C18—C19—C24—C23	−176.77 (15)
C1—C2—C3—C4	−1.1 (3)	C20—C19—C24—C23	0.0 (2)
C2—C3—C4—C5	0.6 (3)	C19—C20—C21—C22	0.0 (2)
C3—C4—C5—C6	0.3 (3)	C20—C21—C22—C23	0.0 (2)
C4—C5—C6—C1	−0.6 (3)	C21—C22—C23—C24	0.0 (2)
C4—C5—C6—C7	178.08 (18)	C22—C23—C24—C19	0.0 (2)

### Hydrogen-bond geometry (Å, º)

Cg1 and Cg2 are the centroids of the C1–C6 and C9–C14 rings, respectively

**Table:**

*D*—H···*A*	*D*—H	H···*A*	*D*···*A*	*D*—H···*A*
C18—H18*A*···*Cg*2^i^	0.97	2.83	3.600 (2)	137
C20—H20···*Cg*1^i^	0.93	2.79	3.642 (2)	153

Symmetry code: (i) −*x*+1, *y*+1/2, −*z*+3/2.

## References

[bb1] Abdoh, M. M. M., Madan Kumar, S., Vinay Kumar, K. S., Manjunath, B. C., Sadashiva, M. P. & Lokanath, N. K. (2013). *Acta Cryst.* E**69**, o17. 10.1107/S1600536812048908 PMC3588308 23476404

[bb2] Al-Qawasmeh, R. A., Lee, Y., Cao, M.-Y., Gu, X., Viau, S., Lightfoot, J., Wright, J. A. & Young, A. H. (2009). *Bioorg. Med. Chem.*, **19** 104–107. 10.1016/j.bmcl.2008.11.001 19027297

[bb3] Bruker (2013). *APEX2*, *SAINT* and *SADABS* Bruker AXS Inc., Madison, Wisconsin, USA.

[bb4] Cremer, D. & Pople, J. A. (1975). *J. Am. Chem. Soc.* **97**, 1354–1358.

[bb5] Macrae, C. F., Bruno, I. J., Chisholm, J. A., Edgington, P. R., McCabe, P., Pidcock, E., Rodriguez-Monge, L., Taylor, R., van de Streek, J. & Wood, P. A. (2008). *J. Appl. Cryst.* **41**, 466–470.

[bb6] Manjunath, B. C., Vinay Kumar, K. S., Madan Kumar, S., Sadashiva, M. P. & Lokanath, N. K. (2013). *Acta Cryst.* E**69**, o1233. 10.1107/S1600536813018412 PMC3793737 24109324

[bb7] Rockliff, B. W. & Davis, E. H. (1966). *Arch. Neurol.* **15**, 129–136. 10.1001/archneur.1966.00470140019003 5329617

[bb8] Sheldrick, G. M. (2008). *Acta Cryst.* A**64**, 112–122. 10.1107/S0108767307043930 18156677

